# Pregnancy and neonatal outcomes among women with early-onset colorectal cancer: a nationwide case–control study

**DOI:** 10.1016/j.eclinm.2023.101963

**Published:** 2023-04-13

**Authors:** Yin Cao, Stephanie Zhao, Tomas S. Bexelius, Jonas Söderling, Mengyao Shi, Bjorn Roelstraete, Barbara B. Warner, Olof Stephansson, Jonas F. Ludvigsson

**Affiliations:** a Division of Public Health Sciences, Department of Surgery, Washington University School of Medicine, St. Louis, MO, USA; b Alvin J. Siteman Cancer Center, Washington University School of Medicine, St. Louis, MO, USA; c Division of Gastroenterology, Department of Medicine, Washington University School of Medicine, St. Louis, MO, USA; d School of Medicine, Washington University School of Medicine, St. Louis, MO, USA; e Department of Women's and Children's Health, Childhood Cancer Research Unit and Department of Medical Epidemiology and Biostatistics, Karolinska Institutet, Stockholm, Sweden; f Department of Paediatric Oncology, Astrid Lindgren Children Hospital, Stockholm, Sweden; g Clinical Epidemiology Division, Department of Medicine, Solna, Karolinska Institutet, Stockholm, Sweden; h Department of Medical Epidemiology and Biostatistics, Karolinska Institutet, Stockholm, Sweden; i Division of Newborn Medicine, Department of Pediatrics, Washington University in St Louis, St Louis, MO, USA; j Division of Women's Health, Department of Obstetrics, Karolinska University Hospital, Stockholm, Sweden; k Department of Paediatrics, Örebro University Hospital, Örebro, Sweden; l Department of Medicine, Columbia University College of Physicians and Surgeons, New York, USA

**Keywords:** Pregnancy, Neonatal, Early-onset, Colorectal cancer

## Abstract

**Background:**

Early-onset colorectal cancer has risen worldwide, leaving more women with colorectal cancer at reproductive ages. We aimed to investigate the risk of adverse pregnancy and neonatal outcomes among women with early-onset colorectal cancer.

**Methods:**

We conducted a nationwide, matched case–control study of maternal/pregnancy outcomes including pre-eclampsia and Cesarean delivery (C-section) as well as neonatal outcomes including preterm birth among 207 births in women with early-onset colorectal cancer (ages 18–49) and 1019 births in women without colorectal cancer in Sweden (1992–2019). Early-onset colorectal cancer cases were identified through the Cancer Register, and outcome data were retrieved through linkage to Medical Birth Register and National Patient Register. Using conditional logistic regression, we estimated multivariable-adjusted odds ratios (ORs) and 95% confidence intervals (CIs).

**Findings:**

Between Jan 1, 1992, and Dec 31, 2019, women with early-onset colorectal cancer who gave birth had increased odds of pre-eclampsia (7.2% *vs* 3.2%; OR = 2.52, 95%CI = 1.25–5.08), any C-section (24.6% *vs* 19.4%; OR = 1.43, 95%CI = 1.00–2.06), particularly emergency C-section (17.4% *vs* 10.5%; OR = 1.79, 95%CI = 1.17–2.75), after adjustment for maternal education level, country of birth, body mass index and smoking in early pregnancy, and comorbidities. Maternal history of early-onset colorectal cancer was also associated with offspring preterm birth (12.1% *vs* 5.2%; OR = 2.31, 95%CI = 1.34–3.99), delineated as spontaneous (OR = 1.06, 95%CI = 0.47–2.39) or medically-indicated preterm birth (OR = 4.48, 95%CI = 2.05–9.79). There was no increased risk of congenital malformation or small for gestational age birth.

**Interpretation:**

In this population-based study, maternal history of early-onset colorectal cancer was associated with risk of both adverse pregnancy (pre-eclampsia, C-section) and neonatal outcomes (preterm birth).

**Funding:**

10.13039/100000002US National Institutes of Health, 10.13039/501100007687Swedish Society of Medicine, 10.13039/100012538Swedish Cancer Foundation.

**Research in context**

**Evidence before this study**

Early-onset colorectal cancer (diagnosed before the age of 50 years) has risen worldwide, leaving more women at reproductive age post-colorectal cancer diagnosis. Studies of pregnancy and neonatal birth outcomes in cancer survivors have been focused on adolescent and young adult cancers and cancers of the female reproductive organs, with little attention given to early-onset colorectal cancer. We searched PubMed using the terms “pregnancy outcomes” and “early-onset colorectal cancer” for English language publications. One study from a single state in Australia reported adverse pregnancy and neonatal outcomes in first post-colorectal cancer pregnancies. However, nationwide studies leveraging matched population-based controls and sibling analyses to minimise the impact of confounding factors are lacking.

**Added value of this study**

In a nationwide case–control study of 207 births in women with early-onset colorectal cancer and 1019 births in matched controls, we discovered a 2.5-fold higher odds of pre-eclampsia and a 79% increased odds of emergency Cesarean delivery in women with early-onset colorectal cancer compared to women without prior colorectal cancer. For neonatal outcomes, maternal history of early-onset colorectal cancer was associated with a 2.3-fold higher odds of preterm birth, which was particularly pronounced for medically-indicated preterm birth. Of note, we found no increased odds of congenital malformations, infant low Apgar score, or small for gestational age birth in women with colorectal cancer. Taken together, our study demonstrates several adverse reproductive outcomes in the growing population of women with early-onset colorectal cancer, providing an important opportunity to inform clinical guidance of this population.

**Implications of all the available evidence**

As the first nationwide study assessing pregnancy and neonatal outcomes in women with early-onset colorectal cancer, our study provides evidence critical for the clinical support of women with early-onset colorectal cancer at reproductive age. Furthermore, this study highlights the need to incorporate pregnancy and neonatal outcomes in cancer survivorship guidelines, which primarily focus on fertility impairment and sexual dysfunction.

## Introduction

Colorectal cancer remains a leading cause of cancer incidence and mortality worldwide. In industrialised nations, a concerning shift in early-onset colorectal cancer epidemiology has emerged, with incidence rates rising in individuals aged 20–49 as the overall incidence of colorectal cancer has stabilised or declined.^1^ Despite a higher prevalence of family history or genetic susceptibility, the majority of early-onset colorectal cancer cases are sporadic and therefore missed by current screening guidelines that begin at age 50 in average-risk individuals.^2^ In a multinational European study, colorectal cancer incidence rates increased by 8.1% annually from 2003 to 2016 in women ages 20–29 and 6.8% annually from 2005 to 2016 in women ages 30–39.^1^ Although the causes of this trend remain unknown, a recent meta-analysis suggests that family history of colorectal cancer, obesity, hyperlipidemia, and alcohol consumption appear to play a role.^3^ Emerging risk factors, such as prolonged sitting,^4^ metabolic syndrome/dysregulation,^5^^,^^6^ and poor diet^7^ including higher intake of sugar-sweetened beverages^8^ are yet to be fully elucidated. With an expanding number of women with colorectal cancer at reproductive age, more attention to fertility and reproductive outcomes in women with early-onset colorectal cancer is crucial for clinical treatment planning and support in this population.^2^

Research on pregnancy and neonatal birth outcomes in cancer survivors has been focused on adolescent and young adult cancers and cancers of the female reproductive organs, reporting increased risk of Cesarean delivery (C-section),^9^ preterm birth,^10^^,^^11^ low birth weight (LBW),^9^^,^^12^ small for gestational age (SGA),^13^ and low Apgar score in offspring.^14^ Outcomes for women with colorectal cancer/early-onset colorectal cancer remain largely unexplored. While female survivors of digestive tract cancers are estimated to be ∼ten percent less likely to give birth than the general population,^15^ studies on pregnancy and neonatal outcomes among women with colorectal cancer are limited. Haggar et al. reported significantly increased risks of ante/post-partum hemorrhage, C-section delivery, infant low Apgar score, and neonatal resuscitation in first pregnancies post-colorectal cancer compared to randomly selected pregnancies in women with no history of any cancer.^14^ However, the study was limited to data from a single state in Australia, which may not be generalisable. To date, nationwide studies leveraging matched population-based controls and sibling analyses to minimise the impact of confounding factors are lacking.

To address these knowledge gaps and guide clinical management, we leveraged data from the Swedish registries to conduct a nationwide case–control study to examine pregnancy and neonatal outcomes among women with early-onset colorectal cancer, comparing with both population-based matched controls and their siblings.

## Methods

### Study design and population

This population-based study leveraged data from multiple national Swedish registers containing validated, prospectively recorded data. The Swedish Cancer Register, established in 1958, has mandatory reporting and captures over 96% of newly detected cancer cases.^16^ The unique Swedish personal identity number was used to link data from the Cancer Register to the Swedish Medical Birth Register (MBR) with data on mode of delivery since 1973 and the National Patient Register (NPR) with data on discharge diagnoses and procedure codes from all hospital admissions since 1964. The MBR covers around 98% of all births in Sweden and contains data from the first antenatal visit until delivery and discharge from the delivery hospital. Completeness of pregnancy and neonatal outcomes in the MBR is bolstered by cross-comparison to the Total Population Register to identify missing records and standardisation of electronic health records across the country, as well as Sweden's universally accessible obstetric care.^17^ This study was approved by the Stockholm Ethics Review Board on August 27, 2014 (No.2014/1287–31/4) and on May 7, 2018 (No.2018/972–32). Informed consent was waived as the study was registry-based.

### Ascertainment of incident early-onset colorectal cancer cases and matched controls

We identified women with a diagnosis of colorectal cancer between age 18 and 49 from 1969 to 2019 in the Swedish Cancer Register, using ICD-7 codes 153 (except 153,4; appendiceal cancer) and 154 (except 154,1; anal cancer) (Table S1). We restricted to women with early-onset colorectal cancer (diagnosed between age 18 to 49) who gave birth between 1992 and 2019, and matched their deliveries with up to five control births from the general population, based on maternal age at delivery (continuous), calendar year of delivery (continuous), maternal parity (nulliparous, parous), and county of residence (categorical). Births in women with early-onset colorectal cancer were restricted to delivery dates after colorectal cancer diagnosis. For parous women (one or more previous births) with early-onset colorectal cancer, only pregnancies after diagnosis were included in this study. Similarly, births in matched controls were restricted to deliveries after colorectal cancer diagnosis of the matched case (index date); for parous women without colorectal cancer, only births after the index date were included in this study. Information on the date of diagnosis (index date for matched controls) and date of delivery were retrieved from the Swedish Cancer Register and MBR. All eligible births in Sweden between Jan 1, 1992, and Dec 31, 2019 were included in the study.

To control for genetic susceptibility and shared childhood exposures, we also compared pregnancy and neonatal outcomes between births in women with early-onset colorectal cancer to births in their female siblings. We identified siblings through the Swedish Multi-generation Register, which contains data on all individuals born in 1932 or later and registered as Swedish residents in 1961 or later.^18^ We restricted the sibling comparators to siblings without a past history of colorectal cancer.

### Assessment of maternal risk factors, and pregnancy and neonatal outcomes

Maternal and pregnancy-related factors were retrieved from the MBR, including maternal country of birth, civil status (i.e. living with a partner), and body mass index (BMI) and smoking status in early pregnancy, while maternal level of education was attained from the Longitudinal Integrated Database for Health Insurance and Labour Market Studies (LISA). We leveraged the National Patient Register (NPR) to retrieve information on diagnoses of maternal comorbidities within five years before the start of pregnancy, including diabetes and autoimmune diseases. Autoimmune disease comorbidity encompassed autoimmune thyroid disease, Type 1 diabetes, ulcerative colitis, Crohn's disease, celiac disease, vitiligo, rheumatoid arthritis, and systemic lupus erythematosus (Table S1).

Pregnancy outcomes were retrieved from the MBR, including induction of labor, C-section (elective or emergency), and instrumental delivery. Maternal outcomes of gestational diabetes and pre-eclampsia were retrieved from both MBR and NPR. Neonatal outcomes were extracted from the MBR, including preterm birth (medically-indicated or spontaneous) and measures of fetal growth including small for gestational age (SGA) and low birth weight (<2500g). Preterm birth (<37 weeks) was classified as either spontaneous onset (preterm labor or preterm premature rupture of membranes) or medically-indicated induction of vaginal or C-section delivery before onset of labor, due to maternal and fetal indications, which can include pre-eclampsia, intrauterine growth restriction, and placental abruption.^19^ Small for gestational age birth was defined as having a birth weight <2 standard deviations or <10th percentile for gestational age, according to Swedish sex-specific reference curves for normal fetal growth.^20^ Additional neonatal outcomes of Apgar score <7 at 5 min, congenital malformations, and neonatal death were also extracted.

### Statistical analyses

Comparing births in women with early-onset colorectal cancer to general population women without prior colorectal cancer, we investigated differences in pregnancy-related outcomes, including gestational diabetes, pre-eclampsia, induction of labor, C-section (elective or emergency), and instrumental delivery. We also examined the odds of adverse neonatal outcomes for offspring, including preterm birth, fetal growth, 5-min Apgar score, congenital malformations, and neonatal death. We then conducted the above analyses compared with pregnancies/births among siblings of women with early-onset colorectal cancer.

Since the study was a matched case–control study, conditional logistic regressions were used to estimate multivariable-adjusted odds ratios (ORs) and 95% confidence intervals (CIs). In addition to conditioning on matching factors (age, calendar year, parity, and county of residence), we adjusted for maternal and pregnancy-related factors: maternal education level (≤9 years, 10–12, >12 years, missing), country of birth (Nordic [Sweden, Denmark, Norway, Finland, Iceland], other/missing), early-pregnancy body mass index (BMI) (<30, ≥30, missing) and smoking status (yes, no, missing) in early pregnancy, and history of diabetes and autoimmune disease. Obesity and smoking are included as shared risk factors for early-onset colorectal cancer^3^ and adverse pregnancy outcomes.^21^ Autoimmune diseases such as systemic lupus erythematosus^22^ and pregestational diabetes mellitus^23^ have also been linked to risk of adverse pregnancy outcomes. Missing values were replaced using a missing indicator. We have also conducted sensitivity analyses using multiple imputation for missing data in BMI (9.7% for cases and 12.3% for controls) and smoking (3.4% for cases and 6.7% for controls) (Table S7). Additional sensitivity analyses were performed with the study population stratified by disease duration/time from index date (<5 years, ≥5 years), anatomic site (colon, rectum), and parity (nulliparous, parous). Due to limited power, we did not test for interactions. No post-hoc analyses were performed. This study follows the Strengthening the Reporting of Observational Studies in Epidemiology (STROBE) guidelines for case–control studies. All analyses were performed in SAS V.9.4 (SAS Institute, Cary, North Carolina, USA).

### Role of the funding source

The funders had no role in study design; the collection, analysis, and interpretation of data; writing of the report; or the decision to submit for publication.

## Results

Between Jan 1, 1992, and Dec 31, 2019, we identified 207 births in 149 women with a history of early-onset colorectal cancer in the Swedish Cancer Register and 1019 births to 1015 controls matched on maternal age, calendar year of delivery, parity, and county of residence (Table 1). The mean (standard deviation) age at colorectal cancer diagnosis among cases was 28.3 (±6.3). The mean maternal age at delivery in both cases and reference women was 33.7 (±4.6) years, and 63% of pregnancies were parous. Women with early-onset colorectal cancer were comparable to their matched controls in terms of country of birth (Nordic *vs* other) and civil status, but they tended to have lower levels of advanced education (>12 years; 43.0% *vs* 53.2%), higher rates of smoking in early pregnancy (9.7% *vs* 6.2%, respectively), and higher rates of autoimmune comorbidity (9.7% *vs* 2.9%).

**Table 1:** Characteristics of women with early-onset colorectal cancer and matched general population reference women giving birth in 1992–2019, Sweden.

Characteristics	Colorectal cancer status	
	Cases (N births = 207)	Controls (N births = 1019)
Women, n	149	1015
Age at colorectal cancer diagnosis, years, mean (SD)	28.3 (6.3)	–
**Matching factors**		
Maternal age at delivery, years, mean (SD)	33.7 (4.6)	33.7 (4.5)
Year of delivery		
1992–1999	33 (15.9%)	163 (16.0%)
2000–2004	33 (15.9%)	162 (15.9%)
2005–2009	59 (28.5%)	295 (28.9%)
2010–2019	82 (39.6%)	399 (39.2%)
Parity^a^		
Nulliparous	77 (37.2%)	373 (36.6%)
Parous	130 (62.8%)	646 (63.4%)
**Maternal characteristics**		
Year of colorectal cancer onset/index date		
1969–1989	24 (11.6%)	118 (11.6%)
1990–1999	38 (18.4%)	187 (18.4%)
2000–2004	58 (28.0%)	288 (28.3%)
2005–2009	49 (23.7%)	236 (23.2%)
2010–2019	38 (18.4%)	190 (18.6%)
Disease duration/time from index date^b^		
<1 year	27 (13.0%)	137 (13.4%)
1–<5 years	98 (47.3%)	484 (47.5%)
5–<10 years	55 (26.6%)	265 (26.0%)
10–<20 years	20 (9.7%)	96 (9.4%)
≥20 years	7 (3.4%)	37 (3.6%)
Maternal country of birth		
Nordic	180 (87.0%)	895 (87.8%)
Other	27 (13.0%)	124 (12.2%)
Civil status of the mother		
Living with a partner	186 (89.9%)	917 (90.0%)
Other/Missing	21 (10.1%)	102 (10.0%)
Level of education		
≤9 years	13 (6.3%)	69 (6.8%)
10–12 years	105 (50.7%)	406 (39.8%)
>12 years	89 (43.0%)	542 (53.2%)
Missing	0	2 (0.2%)
BMI in early pregnancy		
Mean (SD)	25.1 (5.1)	24.8 (4.6)
<18.5	6 (2.9%)	14 (1.4%)
18.5–<25	108 (52.2%)	553 (54.3%)
25–<30	49 (23.7%)	221 (21.7%)
≥30	24 (11.6%)	106 (10.4%)
Missing	20 (9.7%)	125 (12.3%)
Smoking in early pregnancy		
Yes	20 (9.7%)	63 (6.2%)
No	180 (87.0%)	888 (87.1%)
Missing	7 (3.4%)	68 (6.7%)
Comorbidities^c^		
Diabetes	1 (0.5%)	4 (0.4%)
Autoimmune diseases^d^	20 (9.7%)	30 (2.9%)

SD, standard deviation; BMI, body mass index.

a Nulliparous indicates a woman who has never given birth before colorectal cancer diagnosis/index date. Parous indicates a woman who has given birth before diagnosis/index date, but only births after diagnosis/index date are included in the study.

b Time from colorectal cancer diagnosis (index date) to delivery in women with colorectal cancer (matched controls).

c Within five years before the start of pregnancy.

d A full list of autoimmune diseases can be found in Table S1.

Prior history of early-onset colorectal cancer was associated with multiple major pregnancy-related outcomes, including pre-eclampsia, induction of labor, and C-section. Women with early-onset colorectal cancer were more likely to have pre-eclampsia (7.2% *vs* 3.2%; OR 2.52, 95% CI 1.25–5.08) compared to women without colorectal cancer. Although we observed a higher prevalence of gestational diabetes in women with early-onset colorectal cancer (2.4% *vs* 1.6%), the prevalence was low, and we had limited power to evaluate the association. For pregnancy outcomes, we noted that 19.3% of births in women with colorectal cancer involved induction of labor compared to 14% of births in women without colorectal cancer, with an OR of 1.64 (95% CI 1.09–2.46). We also found increased odds of C-section in women with early-onset colorectal cancer (24.6% *vs* 19.4%; OR 1.43, 95% CI 1.00–2.06), which was driven by emergency C-sections rather than elective (17.4% *vs* 10.5%; emergency C-section OR 1.79, 95% CI 1.17–2.75) (Table 2).

**Table 2:** Pregnancy outcomes for women with early-onset colorectal cancer and reference women without prior colorectal cancer.

Outcome	Births in women with colorectal cancer (N = 207)	Births in reference women (N = 1019)	Odds ratio (95% CI)^a^	Odds ratio (95% CI)^b^
**Maternal outcomes**				
Gestational diabetes	5 (2.4%)	16 (1.6%)	1.53 (0.55–4.25)	1.64 (0.51–5.25)
Pre-eclampsia	15 (7.2%)	33 (3.2%)	2.40 (1.27–4.55)	2.52 (1.25–5.08)
**Pregnancy outcomes**				
Induction of labor	40 (19.3%)	143 (14.0%)	1.47 (1.00–2.18)	1.64 (1.09–2.46)
Cesarean delivery	51 (24.6%)	198 (19.4%)	1.37 (0.96–1.96)	1.43 (1.00–2.06)
Elective	15 (7.2%)	91 (8.9%)	0.79 (0.44–1.41)	0.87 (0.48–1.58)
Emergency	36 (17.4%)	107 (10.5%)	1.81 (1.20–2.74)	1.79 (1.17–2.75)
Instrumental delivery	15 (7.2%)	94 (9.2%)	0.73 (0.41–1.31)	0.77 (0.42–1.42)

CI, confidence interval.

a Conditioned on matching set (maternal age, calendar year of delivery, parity, and county of residence).

b Conditioned on matching set and further adjusted for level of education (≤9 years, 10–12, >12 years, missing), country of birth (Nordic, other/missing), body mass index (BMI) (<30, ≥30, missing) and smoking status (yes, no, missing) in early pregnancy, and history of diabetes and autoimmune disease within five years of pregnancy.

History of early-onset colorectal cancer diagnosis was also associated with multiple birth outcomes in the offspring, including preterm birth, very preterm birth, and low birth weight. Out of 206 live births to women with early-onset colorectal cancer, 25 births (12.1%) were preterm, as compared to 53 out of 1016 (5.2%) births to women without prior colorectal cancer. After multivariable adjustment, the odds of preterm birth among women with early-onset colorectal cancer was 2.31 times (95% CI 1.34–3.99) the odds among women without early-onset colorectal cancer (Table 3). This observation was specific to medically-indicated preterm birth (7.3% *vs* 1.9%; OR 4.48, 95% CI 2.05–9.79) rather than spontaneous preterm birth (4.9% *vs* 3.3%; OR 1.06, 95% CI 0.47–2.39). While numbers were limited, we also observed substantially higher rates of very preterm birth, defined as <33 weeks gestation, when comparing births from women with *vs* without prior colorectal cancer (2.4% *vs* 0.4%). Among measurements of fetal growth, we found elevated odds of low birth weight (<2500g; 6.3% *vs* 2.7%; OR 2.95, 95% CI 1.40–6.21) but no association with SGA <10th percentile (7.8% *vs* 8.5%; OR 0.92, 95% CI 0.51–1.69). Although Apgar <7 at 5 min (2.4% *vs* 0.6%) and congenital malformation (5.8% *vs* 4.7%) were more commonly observed in births to women with early-onset colorectal cancer, the associations were not statistically significant.

**Table 3:** Neonatal outcomes for women with early-onset colorectal cancer and reference women without prior colorectal cancer.

Outcome	Births in women with colorectal cancer	Births in reference women	Odds ratio (95% CI)^a^	Odds ratio (95% CI)^b^
**All births, n**	207	1019		
Stillbirth	1 (0.5%)	3 (0.3%)	1.67 (0.17–16.02)	–
**Live births, n**	**206**	**1016**		
**Preterm birth (<37 weeks)**				
Any preterm birth	25 (12.1%)	53 (5.2%)	2.61 (1.56–4.38)	2.31 (1.34–3.99)
Medically-indicated	15 (7.3%)	19 (1.9%)	4.44 (2.15–9.14)	4.48 (2.05–9.79)
Spontaneous	10 (4.9%)	34 (3.3%)	1.45 (0.71–2.95)	1.06 (0.47–2.39)
Very preterm birth^c^	5 (2.4%)	4 (0.4%)	6.25 (1.68–23.27)	6.97 (1.30–37.34)
**Fetal growth**				
SGA <-2SD	6 (2.9%)	17 (1.7%)	1.74 (0.66–4.61)	1.74 (0.53–5.70)
SGA <10th percentile	16 (7.8%)	86 (8.5%)	0.88 (0.50–1.54)	0.92 (0.51–1.69)
Low birth weight (<2500g)	13 (6.3%)	27 (2.7%)	2.54 (1.25–5.15)	2.95 (1.40–6.21)
**Other neonatal outcomes**				
Apgar <7 at 5 min	5 (2.4%)	6 (0.6%)	4.08 (1.25–13.39)	5.47 (0.90–33.27)
Congenital malformations	12 (5.8%)	48 (4.7%)	1.27 (0.66–2.44)	1.30 (0.66–2.57)
Neonatal death	0	1 (0.1%)	–	–

CI, confidence interval; SGA, small for gestational age; SD, standard deviation.

a Conditioned on matching set (maternal age, calendar year of delivery, parity, and county of residence).

b Conditioned on matching set and further adjusted for level of education (≤9 years, 10–12, >12 years, missing), country of birth (Nordic, other/missing), body mass index (BMI) (<30, ≥30, missing) and smoking status (yes, no, missing) in early pregnancy, and history of diabetes and autoimmune disease within five years of pregnancy.

c Very preterm birth was defined as <33 weeks.

When comparing births in women with early-onset colorectal cancer to siblings without colorectal cancer (Table S2), we observed similar trends for the above-mentioned adverse outcomes, especially for higher prevalence of induction of labor (16.7% *vs* 9.0%) (Table S3). We further stratified the main analyses according to time since colorectal cancer diagnosis (<5 *vs* ≥ 5 years), and found that the elevated odds of emergency C-section in pregnancy as well as preterm birth and low birth weight in offspring primarily occurred for births within five years of colorectal cancer diagnosis (Table S4). When stratified according to cancer sites, women with early-onset rectal cancer appeared to have higher absolute and adjusted ORs for induction of labor in pregnancy and offspring preterm birth, compared to women with early-onset colon cancer (Table S5). When stratified by prior history of birth, nulliparous women with early-onset colorectal cancer were more likely to have an emergency C-section compared to controls, while this association was not apparent among parous mothers with early-onset colorectal cancer (Table S6).

## Discussion

In this population-based study of births to women with early-onset colorectal cancer between 1992 and 2019, we identified significantly higher odds of pre-eclampsia in pregnancy and delivery by C-section, compared to offspring of women without a history of colorectal cancer. For neonatal outcomes, we observed elevated odds of preterm birth, very preterm birth, and low birth weight in offspring to mothers with a history of early-onset colorectal cancer. Odds of offspring SGA, Apgar score <7 after 5 min, and congenital malformations were not significantly different between women with early-onset colorectal cancer and general population controls. To our knowledge, this is the first national case–control study of pregnancy and neonatal outcomes in women with early-onset colorectal cancer.

Notably, we found that women with a history of early-onset colorectal cancer were more likely to have induced labor and deliver by C-section, driven by emergency rather than elective C-sections. This is concordant with prior studies in adolescent and young adult cancer survivors that show higher rates of C-section delivery compared to general population controls,^24^ although data distinguishing elective and emergency C-sections are limited.^25^ C-section delivery has been shown to disrupt newborn microbial colonisation compared to vaginal delivery^26^ and likely contributes to later-life diseases such as diabetes and obesity in the offspring,^27^ both of which are associated with higher risk of colorectal cancer. Taken together, our findings raise the potential possibility that pregnancy in women with early-onset colorectal cancer may precipitate transgenerational colorectal cancer risk in the offspring, in addition to their heightened risk from family history of colorectal cancer. As highlighted in recent reviews, there is an unmet need to elucidate transgenerational and early-life risk factors for early-onset colorectal cancer.^3^^,^^28^

Our findings on elevated odds of preterm birth among women with early-onset colorectal cancer are concordant with studies in women with multiple adult cancers,^10^^,^^29^ childhood and adolescent cancers,^11^^,^^30^ and cancers of the female reproductive system.^9^^,^^31^ We also observed increased odds of low birth weight in offspring while odds of SGA birth was not elevated, which suggests that higher rates of low birth weight are likely attributed to preterm birth rather than true impaired intrauterine growth. A valuable addition of our study is the ability to delineate this observation as stemming from medically-indicated rather than spontaneous preterm birth. In fact, we identified elevated odds of pre-eclampsia in pregnant mothers with a history of colorectal cancer, which could serve as a basis for medically-indicated preterm birth, although data on other pregnancy complications were limited. In a sibling analysis, the association of maternal colorectal cancer history and preterm birth was not significant when compared to births in siblings of women with early-onset colorectal cancer, which could also suggest potential confounding from familial or genetic factors. Given the connection to neonatal mortality and later-life chronic diseases,^32^ the risk of preterm birth in offspring remains a serious issue increasingly linked to cancer diagnosis in women of reproductive age.

Although pregnancy outcomes in women with history of childhood/adolescent and female reproductive system cancers are well-studied, the literature connecting colorectal cancer to adverse pregnancy and neonatal outcomes remain limited. In a state-wide study in California of 106 births to women with active colorectal cancer diagnosed during or within one year after pregnancy, Dahling et al. demonstrated higher rates of C-section delivery and preterm birth compared to 4.7 million births in women without colorectal cancer.^33^ However, the majority of cases were diagnosed postpartum and therefore hold less pertinence for management of pregnancy after colorectal cancer diagnosis. In a whole-jurisdiction cohort study in the State of Western Australia, Haggar et al. reported higher risk in women with colorectal cancer (regardless of age at diagnosis) of ante/post-partum hemorrhage, infant low Apgar score, need for neonatal resuscitation, and C-section delivery, but was unable to distinguish between elective and emergency C-sections.^14^ Our study extends existing knowledge by incorporating nationwide register data and elucidating the different causes and indications of preterm birth (medically-indicated vs spontaneous) and C-section delivery (emergency vs elective).

It is likely that our findings of elevated odds of pre-eclampsia, preterm birth, and C-section are related. Pre-eclampsia, particularly pre-eclampsia with severe features, and other pregnancy disorders are often treated by induction of labor regardless of gestational age. Concerning fetal presentation related to these pregnancy disorders may also provide an indication for C-section delivery. While pre-eclampsia and colorectal cancer share obesity as an environmental risk factor (adjusted for as BMI in our analysis), the etiology of pre-eclampsia as a complex, multi-organ disease remains unknown. Our study adjusted for pregestational diabetes within five years of pregnancy, autoimmune comorbidity, and matched on parity, which are established clinical risk factors for pre-eclampsia.^34^ Mechanisms of gut dysbiosis,^35^ endothelial dysfunction, and underlying cellular/metabolic dysregulation^36^ after cancer and anti-cancer therapy may play a role in uteroplacental ischemia leading to pre-eclampsia. An additional analysis excluding deliveries with maternal pre-eclampsia confirmed higher odds of preterm birth (including medically-indicated preterm birth) and emergency C-section in births to women with early-onset colorectal cancer (Table S8). These findings suggest a more complex mechanism in addition to pre-eclampsia pathology for the adverse pregnancy and neonatal outcomes in patients with early-onset colorectal cancer.

With increasing number of early-onset colorectal cancer cases, specialised reproductive and long-term counseling for mothers with early-onset colorectal cancer and their offspring are needed.^37^ At present, counseling on risks of sexual dysfunction and infertility after anticancer therapy are incorporated into American guidelines for colon and rectal cancer survivorship, yet there is a lack of mention of reproductive outcomes in women with history of colorectal cancer and its impact on family planning decisions.^38^^,^^39^ Furthermore, although not specifically studied in patients with colorectal cancer, there is likely a disconnect between guidelines and implementation of clinical fertility discussions, as shown in adolescent and young adult cancer survivors.^40^ While European guidelines address the need for fertility preservation and attention to post-cancer pregnancies, colorectal cancer-specific guidelines do not address reproductive outcomes, likely due to lack of research.^41^^,^^42^ Clinical management should be increasingly tailored to challenges faced by younger patients with colorectal cancer, and more research is needed on reproductive outcomes in this population.

Notable strengths of our study include the nationwide design and use of prospectively-collected, register-based data to avoid recall bias. In addition to matching factors, we also controlled for maternal and pregnancy-related factors such as early pregnancy BMI and smoking status, as well as maternal comorbidities. Furthermore, the analysis of birth data for siblings of women with early-onset colorectal cancer helps to exclude residual confounding from complex environmental and genetic factors. There are also several limitations to this study. First, due to constraints in register-collected data, this study lacked cancer stage and treatment information. Indeed, studies have posited a stronger relationship between radiotherapy exposure and adverse pregnancy outcomes compared to chemotherapy.^11^^,^^30^ However, the differential effects of anticancer treatment are more established for gonadotoxicity impairing fertility; therefore more studies are warranted on birth outcomes specifically.^9^ Second, although this study involved nationwide data, there were limited case numbers of certain neonatal and maternal outcomes, particularly in the sibling analysis. Additionally, limited data on alcohol consumption, ethnicity, hyperlipidemia, and physical activity level may have affected our ability to identify confounding relationships. Our sensitivity analysis suggested higher odds of adverse outcomes for pregnancies within five years after diagnosis. It is possible pregnancies within five years after colorectal cancer diagnosis had increased surveillance, or the adverse changes of cancer and cancer treatment are amplified in the few years following diagnosis.^24^ However, our statistical power was limited in this study, and further studies to elucidate mechanisms are needed. Studies in other race/ethnicity groups are also warranted. Factors unique to Sweden at the population or healthcare system level may also affect the generalisability of these results to other countries.

In this population-based study, maternal history of early-onset colorectal cancer was associated with increased risk of pre-eclampsia and C-section, particularly emergency C-section. Among neonatal birth outcomes, there was also higher risk of preterm birth, limited to medically-indicated preterm birth, and no associations with congenital malformations or SGA.

## Contributors

Study concept and design: YC, JS, JFL

Financial support: YC, TSB, JFL

Administrative support: YC, JFL

Provision of study material or patients: JFL

Collection of data: JFL

Data analysis: JS

Data interpretation: YC, SZ, TSB, JS, MS, BR, BBW, OS, JFL

Manuscript writing: YC, SZ

Final approval of manuscript: YC, SZ, TSB, JS, MS, BR, BBW, OS, JFL

Accountable for all aspects of the work: YC, JFL

YC and JFL accessed and verified the underlying data. JFL is responsible for the decision to submit the manuscript.

## Data sharing statement

No data are available.

## Declaration of interests

YC has served as a consultant for Geneoscopy outside the submitted work. BBW has received grants from the US National Institutes of Health (R01NS124767 and R01HD104607). BBW has also received honorarium for symposium support/lecture from the American Academy of Pediatrics Neonatal-Perinatal fellowship symposium. JFL has coordinated a study on behalf of the Swedish IBD quality register (SWIBREG), which received funding from the Janssen corporation. JFL has also received financial support from MSD to develop a paper reviewing national healthcare registers in China. JFL is currently discussing potential research collaboration with Takeda. All others declare no competing interests.

## References

[1] Vuik F.E., Nieuwenburg S.A., Bardou M. (2019). Increasing incidence of colorectal cancer in young adults in Europe over the last 25 years. Gut.

[2] Eng C., Jacome A.A., Agarwal R. (2022). A comprehensive framework for early-onset colorectal cancer research. Lancet Oncol.

[3] O'Sullivan D.E., Sutherland R.L., Town S. (2022). Risk factors for early-onset colorectal cancer: a systematic review and meta-analysis. Clin Gastroenterol Hepatol.

[4] Nguyen L.H., Liu P.H., Zheng X. (2018). Sedentary behaviors, TV viewing time, and risk of young-onset colorectal cancer. JNCI Cancer Spectr.

[5] Jin E.H., Han K., Lee D.H. (2022). Association between metabolic syndrome and the risk of colorectal cancer diagnosed before age 50 Years according to tumor location. Gastroenterology.

[6] Chen H., Zheng X., Zong X. (2021). Metabolic syndrome, metabolic comorbid conditions and risk of early-onset colorectal cancer. Gut.

[7] Zheng X., Hur J., Nguyen L.H. (2021). Comprehensive assessment of diet quality and risk of precursors of early-onset colorectal cancer. J Natl Cancer Inst.

[8] Hur J., Otegbeye E., Joh H.K. (2021). Sugar-sweetened beverage intake in adulthood and adolescence and risk of early-onset colorectal cancer among women. Gut.

[9] Lambertini M., Blondeaux E., Bruzzone M. (2021). Pregnancy after breast cancer: a systematic review and meta-analysis. J Clin Oncol.

[10] Huang W., Sundquist K., Sundquist J., Ji J. (2020). Risk of being born preterm in offspring of cancer survivors: a national cohort study. Front Oncol.

[11] Signorello L.B., Cohen S.S., Bosetti C. (2006). Female survivors of childhood cancer: preterm birth and low birth weight among their children. J Natl Cancer Inst.

[12] Fossa S.D., Magelssen H., Melve K., Jacobsen A.B., Langmark F., Skjaerven R. (2005). Parenthood in survivors after adulthood cancer and perinatal health in their offspring: a preliminary report. J Natl Cancer Inst Monogr.

[13] Green D.M., Sklar C.A., Boice J.D. (2009). Ovarian failure and reproductive outcomes after childhood cancer treatment: results from the Childhood Cancer Survivor Study. J Clin Oncol.

[14] Haggar F., Pereira G., Preen D. (2013). Maternal and neonatal outcomes in pregnancies following colorectal cancer. Surg Endosc.

[15] Hartman M., Liu J., Czene K. (2013). Birth rates among female cancer survivors: a population-based cohort study in Sweden. Cancer.

[16] Barlow L., Westergren K., Holmberg L., Talback M. (2009). The completeness of the Swedish Cancer Register: a sample survey for year 1998. Acta Oncol.

[17] Cnattingius S., Kallen K., Sandstrom A. (2023). The Swedish medical birth register during five decades: documentation of the content and quality of the register. Eur J Epidemiol.

[18] Ekbom A. (2011). The Swedish multi-generation register. Methods Mol Biol.

[19] Ananth C.V., Vintzileos A.M. (2008). Medically indicated preterm birth: recognizing the importance of the problem. Clin Perinatol.

[20] Marsal K., Persson P.H., Larsen T., Lilja H., Selbing A., Sultan B. (1996). Intrauterine growth curves based on ultrasonically estimated foetal weights. Acta Paediatr.

[21] Creanga A.A., Catalano P.M., Bateman B.T. (2022). Obesity in pregnancy. N Engl J Med.

[22] Smyth A., Oliveira G.H., Lahr B.D., Bailey K.R., Norby S.M., Garovic V.D. (2010). A systematic review and meta-analysis of pregnancy outcomes in patients with systemic lupus erythematosus and lupus nephritis. Clin J Am Soc Nephrol.

[23] Tsakiridis I., Mamopoulos A., Athanasiadis A., Kourtis A., Dagklis T. (2022). Management of pregestational diabetes mellitus: a comparison of guidelines. J Matern Fetal Neonatal Med.

[24] Anderson C., Engel S.M., Mersereau J.E. (2017). Birth outcomes among adolescent and young adult cancer survivors. JAMA Oncol.

[25] Masturzo B., Parpinel G., Macchi C. (2020). Impact of cancer in the management of delivery: 10 years of variations. J Matern Fetal Neonatal Med.

[26] Korpela K., Helve O., Kolho K.L. (2020). Maternal fecal microbiota transplantation in cesarean-born infants rapidly restores normal gut microbial development: a proof-of-concept study. Cell.

[27] Andersen V., Moller S., Jensen P.B., Moller F.T., Green A. (2020). Caesarean delivery and risk of chronic inflammatory diseases (inflammatory bowel disease, rheumatoid arthritis, coeliac disease, and diabetes mellitus): a population based registry study of 2,699,479 births in Denmark during 1973-2016. Clin Epidemiol.

[28] Akimoto N., Ugai T., Zhong R. (2021). Rising incidence of early-onset colorectal cancer - a call to action. Nat Rev Clin Oncol.

[29] Farland L.V., Stern J.E., Hwang S.S. (2021). Early-life cancer, infertility, and risk of adverse pregnancy outcomes: a registry linkage study in Massachusetts. Cancer Causes Control.

[30] van Dorp W., Haupt R., Anderson R.A. (2018). Reproductive function and outcomes in female survivors of childhood, adolescent, and young adult cancer: a review. J Clin Oncol.

[31] Jakobsson M., Gissler M., Sainio S., Paavonen J., Tapper A.M. (2007). Preterm delivery after surgical treatment for cervical intraepithelial neoplasia. Obstet Gynecol.

[32] Chehade H., Simeoni U., Guignard J.P., Boubred F. (2018). Preterm birth: long term cardiovascular and renal consequences. Curr Pediatr Rev.

[33] Dahling M.T., Xing G., Cress R., Danielsen B., Smith L.H. (2009). Pregnancy-associated colon and rectal cancer: perinatal and cancer outcomes. J Matern Fetal Neonatal Med.

[34] Chappell L.C., Cluver C.A., Kingdom J., Tong S. (2021). Pre-eclampsia. Lancet.

[35] Chen X., Li P., Liu M. (2020). Gut dysbiosis induces the development of pre-eclampsia through bacterial translocation. Gut.

[36] Jung E., Romero R., Yeo L. (2022). The etiology of preeclampsia. Am J Obstet Gynecol.

[37] ACOG Committee Opinion No (2018). 747 summary: gynecologic issues in children and adolescent cancer patients and survivors. Obstet Gynecol.

[38] El-Shami K., Oeffinger K.C., Erb N.L. (2015). American cancer society colorectal cancer survivorship care guidelines. CA Cancer J Clin.

[39] Oktay K., Harvey B.E., Partridge A.H. (2018). Fertility preservation in patients with cancer: ASCO clinical practice guideline update. J Clin Oncol.

[40] McKay G.E., Zakas A.L., Osman F., Lee-Miller C., Pophali P., Parkes A. (2021). Disparities between provider assessment and documentation of care needs in the care of adolescent and young adult patients with sarcoma. JCO Oncol Pract.

[41] Argiles G., Tabernero J., Labianca R. (2020). Localised colon cancer: ESMO Clinical Practice Guidelines for diagnosis, treatment and follow-up. Ann Oncol.

[42] Van Cutsem E., Cervantes A., Adam R. (2016). ESMO consensus guidelines for the management of patients with metastatic colorectal cancer. Ann Oncol.

