# Supplementary material for: Pregnancy and neonatal outcomes among women with early-onset colorectal cancer: a nationwide case–control study
Source: eClinicalMedicine. 2023 Apr 13;59:101963. doi: 10.1016/j.eclinm.2023.101963 (PMC10139893; doi:10.1016/j.eclinm.2023.101963)
Supplement: Supplementary material [file mmc1.docx]

**Table S1. ICD codes for colorectal cancer, comorbidities, and outcomes.**

|  | **ICD-7** | **ICD-9**  **(1987-1996)** | **ICD-10**  **(1997-)** |
| --- | --- | --- | --- |
| **Colorectal cancer** | | | |
| Colon cancer | 153 (except 153,4)^a^ |  |  |
| Rectal cancer | 154 (except 154,1)^b^ |  |  |
| **Comorbidities** | | | |
| Diabetes |  | 250 | E10-E14 |
| Autoimmune disease |  |  |  |
| Autoimmune thyroid disease |  | 242A, 242X, 244X, 245C, 245W | E03.5, E03.9, E05.0, E05.5, E05.9, E06.3, E06.5 |
| Type 1 diabetes |  | 250  Restricted to individuals with a first ICD-code at age 30 years or below (<31 years). | E10 |
| Ulcerative colitis |  | 556 | K51 |
| Crohn’s disease |  | 555 | K50 |
| Celiac disease |  | 579A | K90.0 |
| Vitiligo |  | 709A | L80 |
| Rheumatoid arthritis |  | 714 | M05, M06, M12.3 |
| Systemic lupus erythematosus |  | 710A | M32 |
| **Maternal outcomes** | | | |
| Gestational diabetes |  | 648W | O24.4 |
| Pre-eclampsia |  | 642E-642H | O14-O15 |
| Induction of labor |  | 659A, 659B; | O61 |
| **Infant outcomes** | | | |
| Preterm birth |  |  |  |
| Medically indicated: |  |  | Women with preterm premature membrane rupture (O42) were excluded |
| Spontaneous |  |  | O42 |
| **Other neonatal outcomes** | | | |
| Congenital malformation |  | 740-759 | Q00-Q99 |

^a^Appendiceal cancer (ICD-7 153,4) was excluded

^b^Anal cancer (ICD-7 154,1) was excluded

**Table S2. Characteristics of women with early-onset colorectal cancer and their siblings giving birth in 1992-2019.**

| **Characteristics** | **Colorectal cancer status** | | |
| --- | --- | --- | --- |
|  | **Cases**  **(N births = 78)** | | **Siblings without colorectal cancer**  **(N births = 146)** |
| Women, n | 51 | 73 | |
| **Matching factors** |  |  | |
| Maternal age at delivery, years, mean (SD) | 34·1 (4·0) | 30·0 (4·7) | |
| Year of delivery |  |  | |
| 1992 - 1999 | 6 (7·7%) | 38 (26·0%) | |
| 2000 - 2004 | 17 (21·8%) | 27 (18·5%) | |
| 2005 - 2009 | 27 (34·6%) | 35 (24·0%) | |
| 2010 - 2019 | 28 (35·9%) | 46 (31·5%) | |
| Parity^a^ |  |  | |
| Nulliparous | 30 (38·5%) | 59 (40·4%) | |
| Parous | 48 (61·5%) | 87 (59·6%) | |
| **Maternal characteristics** |  |  | |
| Year of colorectal cancer onset |  |  | |
| 1969 - 1989 | 2 (2·6%) | - | |
| 1990 - 1999 | 13 (16·7%) | - | |
| 2000 - 2004 | 31 (39·7%) | - | |
| 2005 - 2009 | 22 (28·2%) | - | |
| 2010 - 2019 | 10 (12·8%) | - | |
| Maternal country of birth |  |  | |
| Nordic | 75 (96·2%) | 145 (99·3%) | |
| Other | 3 (3·8%) | 1 (0·7%) | |
| Civil status of the mother |  |  | |
| Living with a partner | 71 (91·0%) | 134 (91·8%) | |
| Other/Missing | 7 (9·0%) | 12 (8·2%) | |
| Level of education |  |  | |
| ≤9 years | 3 (3·8%) | 22 (15·1%) | |
| 10 - 12 years | 33 (42·3%) | 46 (31·5%) | |
| >12 years | 42 (53·8%) | 78 (53·4%) | |
| BMI in early pregnancy, mean (SD) | 24·6 (4·7) | 23·8 (3·7) | |
| Smoking in early pregnancy |  |  | |
| Yes | 14 (17·9%) | 24 (16·4%) | |
| No | 61 (78·2%) | 115 (78·8%) | |
| Missing | 3 (3·8%) | 7 (4·8%) | |
| Comorbidities^b^ |  |  | |
| Diabetes | 0 | 0 | |
| Autoimmune diseases^c^ | 10 (12·8%) | 1 (0·7%) | |

Abbreviations: SD: standard deviation; BMI, body mass index.

^a^Nulliparous indicates a woman who has never given birth before colorectal cancer diagnosis/index date. Parous indicates a woman who has given birth before diagnosis/index date, but only births after diagnosis/index date are included in the study.

^b^Within five years before the start of pregnancy.

^c^A full list of autoimmune diseases can be found in Table S1.

**Table S3. Pregnancy and neonatal outcomes for women with early-onset colorectal cancer and their siblings**

| **Outcome** | **Births in women with colorectal cancer**  **(N = 78)** | **Births in**  **siblings**  **(N = 145)** | **Odds ratio**  **(95% CI) ^a^** | **Odds ratio**  **(95% CI) ^b^** |
| --- | --- | --- | --- | --- |
| **Pregnancy outcomes** |  |  |  |  |
| Pre-eclampsia | 6 (7·7%) | 4 (2·7%) | 3·06 (0·71-13·20) | 9·79 (0·71-134·33) |
| Induction of labor | 13 (16·7%) | 13 (9·0%) | 2·58 (1·03-6·49) | 7·04 (1·38-35·86) |
| Cesarean section | 19 (24·4%) | 28 (19·3%) | 1·31 (0·60-2·89) | 0·71 (0·21-2·43) |
| Elective | 7 (9·0%) | 9 (6·2%) | 1·21 (0·42-3·51) | 1·34 (0·27-6·64) |
| Emergency | 12 (15·4%) | 19 (13·1%) | 1·29 (0·49-3·43) | 1·20 (0·22-6·54) |
| **Neonatal outcomes** |  |  |  |  |
| Preterm birth (<37 weeks) | 9 (11·5%) | 14 (9·7%) | 1·47 (0·52-4·17) | 0·95 (0·15-6·13) |
| Low birth weight (<2500g)^c^ | 5 (6·4%) | 6 (4·2%) | 1·51 (0·34-6·79) | - |

Abbreviations: CI, confidence interval.

^a^Conditioned on matching set (family).

^b^Conditioned on matching set and further adjusted for maternal age (continuous), calendar year of delivery (continuous), maternal level of education (≤9 years, 10-12, >12 years, missing), country of birth (Nordic, other/missing), body mass index (BMI) (<30, ≥30, missing) and smoking status (yes, no, missing) in early pregnancy, and history of diabetes and autoimmune disease within five years of pregnancy.

^c^Due to limited statistical power, we were unable to conduct the analysis adjusting for covariates for the outcome of low birth weight.

**Table S4. Pregnancy and neonatal outcomes for women with early-onset colorectal cancer and reference women without prior colorectal cancer, according to disease duration/time from index date**

| **Outcome** | **Births in women with colorectal cancer** | **Births in**  **reference women** | **Odds ratio**  **(95% CI) ^a^** | **Odds ratio**  **(95% CI) ^b^** |
| --- | --- | --- | --- | --- |
| **Disease <5 years** | 124 | 618 |  |  |
| **Pregnancy outcomes** |  |  |  |  |
| Pre-eclampsia | 7 (5·6%) | 24 (3·9%) | 1·50 (0·62-3·63) | 1·44 (0·55-3·74) |
| Induction of labor | 22 (17·6%) | 87 (14·1%) | 1·34 (0·80-2·25) | 1·43 (0·84-2·46) |
| Cesarean section | 37 (29·6%) | 126 (20·4%) | 1·65 (1·07-2·55) | 1·79 (1·14-2·80) |
| Elective | 10 (8·0%) | 57 (9·2%) | 0·84 (0·41-1·72) | 0·96 (0·45-2·04) |
| Emergency | 27 (21·6%) | 69 (11·2%) | 2·21 (1·34-3·66) | 2·24 (1·31-3·81) |
| **Neonatal outcomes** |  |  |  |  |
| Preterm birth (<37 weeks) | 16 (12·9%) | 36 (5·8%) | 2·49 (1·32-4·70) | 2·38 (1·22-4·64) |
| Low birth weight (<2500g) | 10 (8·1%) | 22 (3·6%) | 2·39 (1·08-5·32) | 3·11 (1·31-7·42) |
|  |  |  |  |  |
| **Disease ≥5 years** | 82 | 398 |  |  |
| **Pregnancy outcomes** |  |  |  |  |
| Pre-eclampsia | 8 (9·8%) | 9 (2·3%) | 4·27 (1·57-11·61) | 14·28 (2·56-79·66) |
| Induction of labor | 18 (22·0%) | 56 (14·1%) | 1·77 (0·97-3·22) | 2·19 (1·15-4·17) |
| Cesarean section | 14 (17·1%) | 72 (18·1%) | 0·96 (0·51-1·82) | 0·96 (0·49-1·88) |
| Elective | 5 (6·1%) | 34 (8·5%) | 0·69 (0·26-1·86) | 0·68 (0·24-1·98) |
| Emergency | 9 (11·0%) | 38 (9·5%) | 1·22 (0·57-2·62) | 1·21 (0·54-2·69) |
| **Neonatal outcomes** |  |  |  |  |
| Preterm birth (<37 weeks) | 9 (11·0%) | 17 (4·3%) | 3·34 (1·32-8·45) | 3·65 (0·99-13·54) |
| Low birth weight (<2500g) | 3 (3·7%) | 5 (1·3%) | 4·16 (0·82-21·03) | 3·05 (0·39-23·79) |

Abbreviations: CI, confidence interval.

^a^Conditioned on matching set (maternal age, calendar year of delivery, parity, and county of residence).
^b^Conditioned on matching set and further adjusted for further adjusted for level of education (≤9 years, 10-12, >12 years, missing), country of birth (Nordic, other/missing), body mass index (BMI) (<30, ≥30, missing) and smoking status (yes, no, missing) in early pregnancy, and history of diabetes and autoimmune disease within five years of pregnancy.

**Table S5. Pregnancy and neonatal outcomes for women with early-onset colorectal cancer and reference women without prior colorectal cancer, according to anatomic site**

| **Outcome** | **Births in women with colorectal cancer** | **Births in**  **reference women** | **Odds ratio**  **(95% CI) ^a^** | **Odds ratio**  **(95% CI) ^b^** |
| --- | --- | --- | --- | --- |
| **Colon cancer** | 140 | 688 |  |  |
| **Pregnancy outcomes** |  |  |  |  |
| Pre-eclampsia | 11 (7·8%) | 22 (3·2%) | 2·70 (1·25-5·84) | 2·92 (1·27-6·70) |
| Induction of labor | 24 (17·0%) | 90 (13·1%) | 1·38 (0·84-2·26) | 1·45 (0·87-2·42) |
| Cesarean section | 36 (25·5%) | 141 (20·5%) | 1·37 (0·89-2·12) | 1·47 (0·94-2·30) |
| Elective | 10 (7·1%) | 58 (8·4%) | 0·83 (0·40-1·69) | 1·00 (0·47-2·11) |
| Emergency | 26 (18·4%) | 83 (12·1%) | 1·68 (1·03-2·74) | 1·75 (1·05-2·91) |
| **Neonatal outcomes** |  |  |  |  |
| Preterm birth (<37 weeks) | 14 (10·0%) | 39 (5·7%) | 1·93 (0·99-3·74) | 1·87 (0·92-3·79) |
| Low birth weight (<2500g) | 8 (5·7%) | 18 (2·6%) | 2·27 (0·94-5·45) | 2·88 (1·11-7·52) |
|  |  |  |  |  |
| **Rectal cancer** | 66 | 328 |  |  |
| **Pregnancy outcomes** |  |  |  |  |
| Pre-eclampsia | 4 (6·1%) | 11 (3·4%) | 1·86 (0·58-5·97) | 1·27 (0·25-6·38) |
| Induction of labor | 16 (24·2%) | 53 (16·2%) | 1·66 (0·87-3·15) | 2·14 (1·02-4·50) |
| Cesarean section | 15 (22·7%) | 57 (17·4%) | 1·37 (0·73-2·56) | 1·27 (0·63-2·57) |
| Elective | 5 (7·6%) | 33 (10·1%) | 0·73 (0·28-1·93) | 0·62 (0·19-2·07) |
| Emergency | 10 (15·2%) | 24 (7·3%) | 2·19 (1·01-4·72) | 1·70 (0·68-4·24) |
| **Neonatal outcomes** |  |  |  |  |
| Preterm birth (<37 weeks) | 11 (16·7%) | 14 (4·3%) | 4·44 (1·90-10·33) | 3·67 (1·24-10·85) |
| Low birth weight (<2500g) | 5 (7·6%) | 9 (2·8%) | 3·17 (0·95-10·65) | 5·03 (0·94-26·82) |

Abbreviations: CI, confidence interval.

^a^Conditioned on matching set (maternal age, calendar year of delivery, parity, and county of residence).
^b^Conditioned on matching set and further adjusted for level of education (≤9 years, 10-12, >12 years, missing), country of birth (Nordic, other/missing), body mass index (BMI) (<30, ≥30, missing) and smoking status (yes, no, missing) in early pregnancy, and history of diabetes and autoimmune disease within five years of pregnancy.

**Table S6. Pregnancy and neonatal outcomes for women with early-onset colorectal cancer and reference women without prior colorectal cancer, according to parity**

| **Outcome** | **Births in women with colorectal cancer** | **Births in**  **reference women** | **Odds ratio**  **(95% CI) ^a^** | **Odds ratio**  **(95% CI) ^b^** |
| --- | --- | --- | --- | --- |
| **Nulliparous** | 77 | 373 |  |  |
| **Pregnancy outcomes** |  |  |  |  |
| Pre-eclampsia | 9 (11·7%) | 19 (5·1%) | 2·58 (1·10-6·06) | 4·68 (1·47-14·90) |
| Induction of labor | 18 (23·4%) | 67 (18·0%) | 1·42 (0·77-2·60) | 1·55 (0·81-2·94) |
| Cesarean section | 24 (31·2%) | 86 (23·1%) | 1·52 (0·89-2·63) | 1·59 (0·91-2·78) |
| Elective | 9 (11·7%) | 58 (15·5%) | 0·72 (0·34-1·53) | 0·83 (0·38-1·82) |
| Emergency | 15 (19·5%) | 28 (7·5%) | 3·01 (1·50-6·04) | 3·06 (1·46-6·42) |
| **Neonatal outcomes** |  |  |  |  |
| Preterm birth (<37 weeks) | 12 (15·6%) | 25 (6·7%) | 2·61 (1·23-5·54) | 2·18 (0·90-5·32) |
| Low birth weight (<2500g) | 7 (9·1%) | 15 (4·0%) | 2·41 (0·93-6·26) | 3·10 (1·10-8·70) |
|  |  |  |  |  |
| **Parous** | 129 | 643 |  |  |
| **Pregnancy outcomes** |  |  |  |  |
| Pre-eclampsia | 6 (4·6%) | 14 (2·2%) | 2·19 (0·83-5·79) | 2·67 (0·87-8·14) |
| Induction of labor | 22 (16·9%) | 76 (11·8%) | 1·51 (0·91-2·52) | 1·57 (0·92-2·68) |
| Cesarean section | 27 (20·8%) | 112 (17·4%) | 1·26 (0·79-2·03) | 1·25 (0·77-2·05) |
| Elective | 6 (4·6%) | 33 (5·1%) | 0·90 (0·37-2·21) | 0·86 (0·33-2·24) |
| Emergency | 21 (16·2%) | 79 (12·3%) | 1·39 (0·83-2·35) | 1·30 (0·75-2·24) |
| **Neonatal outcomes** |  |  |  |  |
| Preterm birth (<37 weeks) | 13 (10·1%) | 28 (4·4%) | 2·62 (1·29-5·32) | 2·49 (1·18-5·28) |
| Low birth weight (<2500g) | 6 (4·7%) | 12 (1·9%) | 2·71 (0·95-7·76) | 3·62 (1·06-12·38) |

Abbreviations: CI, confidence interval.

^a^Conditioned on matching set (maternal age, calendar year of delivery, parity, and county of residence).
^b^Conditioned on matching set and further adjusted for level of education (≤9 years, 10-12, >12 years, missing), country of birth (Nordic, other/missing), body mass index (BMI) (<30, ≥30, missing) and smoking status (yes, no, missing) in early pregnancy, and history of diabetes and autoimmune disease within five years of pregnancy.

**Table S7. Pregnancy and neonatal outcomes for women with early-onset colorectal cancer and reference women without prior colorectal cancer, after multiple imputation for BMI (continuous) and smoking (yes, no)**

| **Outcome** | **Births in women with colorectal cancer**  **(N = 207)** | **Births in**  **reference women**  **(N = 1019)** | **Odds ratio**  **(95% CI) ^a^** | **Odds ratio**  **(95% CI) ^b^** |
| --- | --- | --- | --- | --- |
| **Pregnancy outcomes** |  |  |  |  |
| Pre-eclampsia | 15 (7·2%) | 33 (3·2%) | 2·40 (1·27-4·55) | 2·65 (1·27-5·54) |
| Induction of labor | 40 (19·3%) | 143 (14·0%) | 1·47 (1·00-2·18) | 1·52 (0·98-2·33) |
| Cesarean section | 51 (24·6%) | 198 (19·4%) | 1·37 (0·96-1·96) | 1·52 (1·03-2·24) |
| Elective | 15 (7·2%) | 91 (8·9%) | 0·79 (0·44-1·41) | 0·83 (0·44-1·55) |
| Emergency | 36 (17·4%) | 107 (10·5%) | 1·81 (1·20-2·74) | 1·99 (1·26-3·15) |
| **Neonatal outcomes** |  |  |  |  |
| Preterm birth (<37 weeks) | 25 (12·1%) | 53 (5·2%) | 2·61 (1·56-4·38) | 2·42 (1·35-4·33) |
| Low birth weight (<2500g) | 13 (6·3%) | 27 (2·7%) | 2·54 (1·25-5·15) | 3·43 (1·57-7·47) |

Abbreviations: BMI, body mass index; CI, confidence interval.

^a^Conditioned on matching set (maternal age, calendar year of delivery, parity, and county of residence).

^b^Conditioned on matching set and further adjusted for maternal age (continuous), calendar year of delivery (continuous), maternal level of education (≤9 years, 10-12, >12 years, missing), country of birth (Nordic, other/missing), body mass index (BMI) (continuous) and smoking status (yes, no, missing) in early pregnancy, and history of diabetes and autoimmune disease within five years of pregnancy.

**Table S8. Pregnancy and neonatal outcomes for women with early-onset colorectal cancer and reference women without prior colorectal cancer, excluding deliveries with maternal pre-eclampsia**

| **Outcome** | **Births in women with colorectal cancer**  **(N = 191)** | **Births in**  **siblings**  **(N = 984)** | **Odds ratio**  **(95% CI) ^a^** | **Odds ratio**  **(95% CI) ^b^** |
| --- | --- | --- | --- | --- |
| **Pregnancy outcomes** |  |  |  |  |
| Pre-eclampsia | - | - | - | - |
| Induction of labor | 33 (17·3%) | 120 (12·2%) | 1·58 (1·03-2·41) | 1·73 (1·11-2·70) |
| Cesarean section | 44 (23·0%) | 183 (18·6%) | 1·33 (0·91-1·94) | 1·37 (0·93-2·02) |
| Elective | 12 (6·3%) | 83 (8·4%) | 0·75 (0·40-1·41) | 0·81 (0·42-1·56) |
| Emergency | 32 (16·8%) | 100 (10·2%) | 1·76 (1·14-2·73) | 1·74 (1·11-2·75) |
| **Neonatal outcomes** |  |  |  |  |
| Preterm birth (<37 weeks) | 22 (11·5%) | 44 (4·5%) | 2·68 (1·55-4·65) | 2·36 (1·32-4·25) |
| Low birth weight (<2500g) | 10 (5·2%) | 20 (2·0%) | 2·78 (1·23-6·25) | 3·78 (1·54-9·27) |

Abbreviations: CI, confidence interval.

^a^Conditioned on matching set (maternal age, calendar year of delivery, parity, and county of residence).

^b^Conditioned on matching set and further adjusted for maternal age (continuous), calendar year of delivery (continuous), maternal level of education (≤9 years, 10-12, >12 years, missing), country of birth (Nordic, other/missing), body mass index (BMI) (<30, ≥30, missing) and smoking status (yes, no, missing) in early pregnancy, and history of diabetes and autoimmune disease within five years of pregnancy.

**Text**. **Methods for identification of cases and controls and matching process.**

We first identified patients with colorectal cancer from the Swedish Cancer Register (excluding appendiceal and anal cancers) diagnosed between ages 18-49. Then, we identified corresponding births of women with colorectal cancer in the Swedish Medical Birth Register, limiting to deliveries between 1992-2019 and to deliveries after early-onset colorectal cancer diagnosis date. For control deliveries, we restricted to births in the general population between 1992-2019. Then, matching was performed based on maternal age at delivery, calendar year of delivery, maternal parity, and county of residence. Control births were restricted to those after index date (corresponding to diagnosis date for women with early-onset colorectal cancer) and to mothers without history of colorectal cancer diagnosis.
